# Examining Governing Board Functions and Health Center Performances During Health System Reform: A Cross-sectional Study in 4 Regional States of Ethiopia

**DOI:** 10.34172/ijhpm.2020.235

**Published:** 2020-12-02

**Authors:** Mesele Damte Argaw, Binyam Fekadu Desta

**Affiliations:** USAID Transform: Primary Health Care, JSI Research & Training Institute Inc., Addis Ababa, Ethiopia.

**Keywords:** Governance, Governing Board, Health Center, Reform, Ethiopia

## Abstract

**Background:** Since 1995, the Ethiopian health system has been managed through decentralizing functions, resources, and authorities to local levels. As a result, health centers are led and managed by governing boards. In addition, the national health system strives to transform the performance of health centers through the implementation of reforms. Therefore, this study aims to examine the relationship between governing board functions and health center performances within a health reform context in 4 agrarian regions of Ethiopia.

**Methods:** A cross-sectional survey was conducted from August 28, 2018 to September 30, 2018. Primary data were collected from governing board chairpersons or their designees using interviewer-administered structured questionnaires. The performance of each health center was rated out of 100 percentage points against the Ethiopian Health Center Reform Guideline (EHCRIG) standards. Secondary data were abstracted from a routine health information database using customized tools to capture achievements on 69 EHCRIG standards and its 174 validation criteria. Since the data violate the assumptions of the parametric test, the Spearman’s rank (rho) correlation test, (a non-parametric test) was employed to see if any correlation exists among 4 parameters; namely: structure, roles and responsibilities, training and development of governing boards, and performance of health centers against EHCRIGs standards. A statistically significant relationship was claimed at *P*<.050.

**Results:** All 83 health center governing boards or designees who were approached for this study, participated. The mean health center governing board function score with standard deviation was 56.0% (SD ± 14.5%). The overall performance of health centers against EHCRIGs was 70.4% (SD ± 15.0%). There was a statistically significant and strong correlation (Spearman rho correlation coefficient) between health center performance scores measured against reform standards with governing board scores of (rho=0.866, *P*<.001) and overall governance scores (rho=0.828, *P*<.001).

**Conclusion:** Based on the results of this study, we can conclude that well-functioning health center governing boards can improve the performance of health centers against clinical, and management reform standards. Therefore, continuous strengthening of the capacity of governing boards, focusing on improving implementation of their roles and responsibilities, and continuing training on business management is recommended.

## Background

**Key Messages**

** Implications for policy makers**

- This study highlights the need to capacitate governing boards to execute their function or roles and responsibilities for higher health center performances against the reform standards.
- In decentralized health systems, health center governing boards of lower or upper-middle-income countries are an ideal structure to allocate resources as well as bridge performance and quality improvement gaps. This study reveals areas of focus for future directions and support by policy-makers and program implementers to achieve better health outcomes.
- With regards to health system strengthening interventions, this study documents the importance of measurements against minimum health reform standards, generation of information from data and institutionalization of tools, and platforms for following up health center governing boards. Therefore, the results of this study fill gaps in scientific literature on the relationship between the function of governing boards and the performance of health centers against reforms.

** Implications for the public**

In a decentralized health system, communication on the structure, as well as the roles and responsibilities of governing boards helps the public and community members to demand their rights on issues concerning the leading, managing and governing practices of the health services.

Globally, it is a common strategy and practice to assign leadership and management roles to facility governing boards. The main duties of governing boards include oversight of external communication, accountability to community members, and close follow up of the performance of non-profit health facilities.^1^ Well-functioning governing boards have a direct, positive effect on performances of organizations.^2,3^

 The Ethiopian health sector strategic plan (2015-2020) aims to ensure access to equity and quality of essential primary healthcare services.^4^ The health service delivery system in the country is organized into 3 levels; namely: primary, secondary and tertiary.^5^ Health centers operate at the primary level of the health system, where the community receives promotive, preventive, curative, and rehabilitative outpatient care including basic laboratory and pharmacy services with a capacity for 10 beds for emergency and delivery services.^6^

 Ethiopia is a Federal Democratic Republic composed of 9 regional states and 2 city administrations. These structures are divided into 79 zones, which are further divided into 1000 woredas (districts). A district is a basic decentralized administrative unit that has an administrative council composed of elected members.^4,5^

 Governance of the health system in Ethiopia mirrors the wider context of Ethiopia’s political system^7^ where the Federal Government in consultation with regional states develops policies, strategies, guidelines, and standards. The regional states and districts are empowered to govern their respective levels of health system and steward resources accordingly.^7-9^ All health centers, as part of the primary health tier system, are managed and directed by their own governing boards. The boards are intended to create empowerment and autonomy in providing a good level of care, accountability for services rendered, and responsiveness to community demands. In addition, it is their duty to facilitate the mobilization, allocation, and utilization of resources, as necessary, to run basic health services. As a result, every health center is enabled to deliver effective and efficient essential health services to its catchment population.^6,10^

 In 2016, the Federal Ministry of Health developed the Ethiopian Health Center Reform Implementation Guidelines (EHCRIGs)^[1]^ – a set of minimum management and clinical standards – that all health centers are required to adhere to.^11^ The guidelines contain 81 health center management and clinical standards that assess ten functions presented in chapters. These are: leadership and governance, health center and health post linkage, patient flow management, medical records management, pharmacy services, laboratory services, clean and safe health facilities, medical equipment management and biomedical engineering, human resource management, and quality improvement and health information system management.^11,12^

 The leadership and governance chapter is dedicated to improving the unique set of skills for governing boards, in terms of managing their organization and liaising with external agencies and the local community.^11^ In addition, the governing board is expected to lead their respective organization through changes; identifying, and solving any challenges that arise. This chapter has twelve minimum standards with 35 validation composite criteria whereby compliance by health centers will enable them to improve service delivery, transparency, accountability, and responsiveness to community needs and demands.^11^

 Despite having implemented the EHCRIGs for over 4 years, there has been little evidence gathered on the function of health center governing boards and their link with performance average scores against reform minimum standards. Therefore, this study aims to assess the relationship between the functions of governing boards and the performance of health centers in meeting minimum reform standards in 4 agrarian regions of Ethiopia.

## Methods

###  Study Site

**Figure 1:**
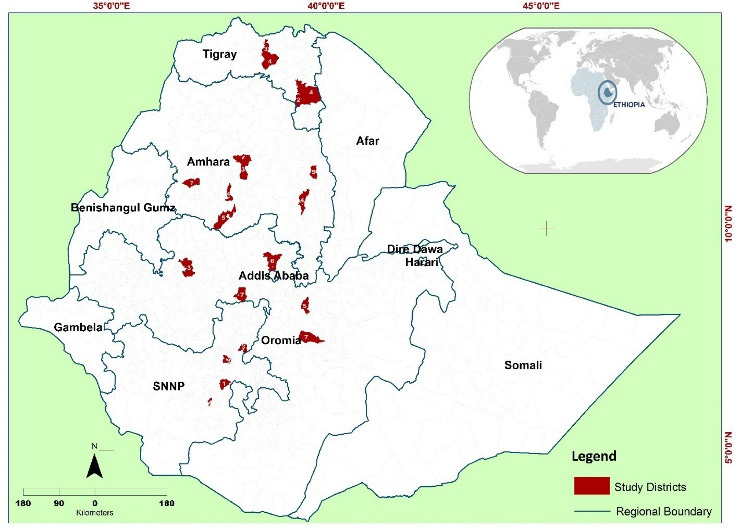
Map of Ethiopia by Regions and Study Sites, 2018. The map shows the location of Ethiopia in Africa. In addition, it presents the regional states and targeted study sites.

 Ethiopia is located in the horn of Africa. The land area is estimated to be about 1.1million square kilometers.^13^ United States Agency for International Development (USAID)Transform: Primary Health Care^[2]^ provides technical support on health system strengthening to 1837 health centers, 9510 health posts, and 117 primary hospitals located in 400 districts, within 4 regional states of Ethiopia.^10^ This study was conducted in 28, 28, 14 and 13 health centers located in Amhara, Oromia, SNNP (Southern Nations, Nationalities, and People) and Tigray regions, respectively (Figure 1). The investigators developed a map using the open data shapefiles of the Ethiopian Central Statistics Agency (2016), (https://africaopendata.org/dataset/ethiopia-shapefiles). All health centers, have established governing boards^[3]^ who oversee the activities of the health center, and are accountable to the district council and catchment community members. Regional health service delivery administrative directives recommend that governing boards should be constituted of 7 to 9 members and include representatives from public institutions, (ie, women and children’s affairs, finance and economy, education, health and woreda administration offices), community members, and health center staff, demonstrating a gender balance among members.^10^ Health center governing board members are officially assigned for 3 to 6 years by the head of the district council or mayor of the city. The USAID Transform Primary Healthcare Activity initiated support to 83 targeted health centers through the orientation of 1306 health center staff and 3600 governing board members, on their duties, responsibilities and the minimum standards of the EHCRIGs.^12^ The average catchment population for each of these health centers is 26 365.

###  Study Design

 A cross-sectional study design using a quantitative approach was conducted in 4 regional states of Ethiopia. This observational study design enabled the investigators to collect and examine data at one specific point in time. Hence, the exposures and outcome variables can be measured at the same time.^14^

###  Analytical Framework

 The investigators adapted a healthcare performance intelligence framework.^15-17^ In order to uncover the relationship between the function of governing boards and the performance of health centers, a hierarchical structure of the performance intelligence framework best fit the process of analyzing the data. The first step deals with performance measurements that include the process of healthcare big data transformation into reliable and valid indicators. The second step is relates to healthcare governance and management practices that enable the inferring of information obtained from indicators into knowledge. The third step deals with utilization, upon which all stakeholders can act.^15-17^

###  Sample Size and Sampling 

 USAID Transform: Primary Healthcare initiated technical support on the implementation of ECHRIGs and capacitated the leadership and governing bodies of 83 health centers. The sampling frame used in this study was developed form the Activity’s reports.^18^ All targeted health centers were purposively selected and enrolled in this study.

###  Data Collection and Data Quality 

 Data collection tools, questionnaires, and data extraction forms were developed after reviewing relevant literature and nationally endorsed validation checklists (Supplementary file 1).^11,12,18,19^ Both primary and secondary data were used in this study. The primary data collection tool contained 23 semi-structured questions, grouped into 3 categories. The first parameter posed 4 questions which were dedicated to measuring the structure of governing boards, the second parameter posed 15 questions designed to measure the roles and responsibilities of health center governing boards, and the third parameter asked 4 questions intending to measure training and development-related factors. The performance of each health center was rated out of 100 percentage points against the EHCRIG standards. Secondary data were abstracted from a routine health information database using customized, nationally recommended tools to capture achievements on 69 EHCRIG minimum standards containing 174 validation composite criteria.^11,12^ A detailed description is presented in Table 1.

**Table 1:** Description of Standards and Indicators^11,12^

**Chapter **	**Description of the Indicators and Composite Criteria Considered**
Health center and health post linkage	This chapter deals with scores that measure health center and health post linkages. The standards include: availability of health center and health post linkage guidelines, assigned focal persons, agreed upon action plans with budgets, establishment of women development groups and a 1 to 5 network of health development army groups, weekly facilitation of supportive supervisions, monthly capacity enhancement and performance review meetings, and availability of tracer drugs in health posts (8 standards with 30 validation criteria).
Patient flow and service organization	The patient flow and service organization chapter has 6 standards and 20 validation criteria. The details of this standard include: developed protocol and procedures on patient flow, triage, referral and preparations to manage emergency services, training of staff to implement protocols, equipping of liaison officers, telephone facilities, registers, books, referral directories, appointment registers, clear labels and/or signage of facilities and services, establishment of maternal homes (waiting areas) with full sets of facilities ie, showers, toilets, kitchens etc (6 standards with 20 validation criteria).
Medical records management	The medical records management chapter deals with the establishment of well-organized archive rooms, institutionalization of unique patient identifiers, establishment of digital or manual indexes and availability of essential resources like cards forms, printed sheets, confidentiality of patient information through protocol and process, compliance officers, creating staff awareness on developed processes and arranging an experience sharing event (4 standards with 9 validation criteria).
Pharmacy services	The pharmacy service chapter is designed to meet the primary needs of all customers. The detailed criteria includes establishment of a drug and therapeutic committee with Terms of References, developed annual plans, testimony with minutes and reports, pharmacy department led by a pharmacist and stores managed by a diploma level pharmacy technician, development of facility level drug list using VEN categorization, forecasting, purchasing and disposal guidelines, dispensing of drugs after recording detailed information of patients and drugs, establishment of a drug information center, protocol for management of side effects and other problems, use of logistical management tools, inventory of drugs and disposal guidelines, availability of tracer drugs in health posts, preparation of standard rooms, and an audit report (13 standards with 21 validation criteria).
Laboratory services	This chapter deals with the provision of laboratory services. Some of the standards refer to the availability of public information about the services, time, and costs, counseling services with clients who understand the meaning of investigations and results, preparation of adequate rooms, assignment of human resources and supplies as per the requirements and standards, institutionalization of laboratory operating management systems, availability of SOP on sample collections, transport, storing and disposals, safety procedures, diagnostic algorisms, laboratory information systems, maintenance procedures of lab equipment, safety procedures in place eg, fire extinguisher and trained staff on safety, data safety and confidentiality, and internal quality control and participation in external quality assurance (9 standards with 23 validation criteria).
Clean, and safe health facility	These standards measure the health facility’s engagement and dedication to providing services in a clean, safe and healthy environment, assignment of a focal person to facilitate technical support to all staff, allocation of budget, institutionalization of a functional infection prevention committee using minutes, action plans and feedback, and availability of personal protective equipment such as soaps, detergents, mops and hand tools to prepare land for gardening, support to health posts in implementing standard infection prevention principles in their facilities, all staff training on infection prevention, health education to clients and patients on infection prevention, following of procedures of waste management, and adherence to medical instrument decontamination and sterilization procedures (10 standards with 13 validation criteria).
Medical equipment management and biomedical engineering	The biomedical engineering chapter deals with the availability of functional medical equipment in health facilities. Some of the standards are inventory of medical equipment, functionality of new equipment, orientation to staff on use and care of new equipment, preparation and maintaining of request forms, maintenance of equipment, functionality and safety of medical equipment, availability of water and electricity supply for 24 hours a day/7 days week (8 standards with 40 validation criteria).
Human resources management	The human resource chapter deals with human resource management and development. The standards are: availability of human resource personnel, archiving of personal files with job descriptions, employment letter and other testimonies, human resource development plans, establishment of motivation and reward systems, appraisal of the performance of the health workforce every 6 months and use of uniform and identification cards by all staff (6 standards with 8 validation criteria).
Quality improvement and health information system management	This chapter deals with quality improvement and routine health information system requirements. The standards are: establishment of quality improvement teams (minutes, Term of References), sharing of workplans with departments and staff aggregated by weeks, months and quarters, implementation of quality improvement tools in selected health services i.e. problem-solving tools, adherence to routine health information system requirements, timely, complete and consistent report submission and use of data for decision-making, engagement of the community towards quality improvement, and completion of client satisfaction and other surveys (5 standards with 10 validation criteria).

Abbreviations: SOP, standard operating procedures; VEN, vital, essential and non-essential.

 The questionnaires were prepared in English and were translated into Amharic, the official national language, then translated back to English to check consistency of the tools. Before the actual data were collected, a two-day training of 8 data collectors and 4 supervisors took place. In addition, a pilot testing was arranged in 8 health centers in all 4 regions. The principal investigators facilitated the training and pilot testing that covered ethical principles, data collection tools, and interviewing and data extraction techniques. The purpose of the training and pilot testing was to identify questions that were not clear for both the interviewers and interviewees and make the necessary modifications though rephrasing. This process ensured the validity of the actual data.^20^ The results of the pilot test were not included in the final findings of the study.

 Primary data were collected from 83 health center governing board chairpersons or their designees from August 28, 2018 to September 30, 2018 in Amhara, Oromia, Tigray, and SNNP regions. Following that, the average scores on performances of health centers were extracted from the regional EHCRIGs database and were linked with the primary data set. Completeness and consistency of filled questionnaires were checked on a daily basis.

###  Data Analysis 

 The data were manually cleaned and checked for consistency, then entered into the Statistical Package for Social Science Research (SPSS IBM version 20) a computer software program used for analysis.^21^ The results of the descriptive analysis were presented using tables and graphs. To determine the functionalities of health center governing boards, 23 closed-ended questions were rated with a ‘1’ for positive responses and a ‘0’ for negative responses. The sum scores were translated into 100.0 percentage points. The functionality of health center governing boards were then categorized into 3: as functional for scores ≥80%, semi-functional for scores 60.0% to 79.9%, and non-functional for scores <60%. The performance of health centers by regions were measured out of 100% against the EHCRIGs’ 69 standards and 174 validation criteria.^11^ The health centers were categorized using reform performance scores as ‘high’ for scores ≥80%, ‘medium’ for scores 60.0% to 79.9%, and ‘low’ for scores <60%.^8^ Since the data violate the assumptions of a parametric test, a Spearman’s rank (rho) correlation test (a non-parametric test) was employed to see if any correlation exists among the 4 parameters, namely: structure, roles and responsibilities and training development of governing board checklists, and performance of health centers against EHCRIG standards (Supplementary file 2). A statistically significant Spearman’s rank (rho) correlation test was claimed at *P*< .050.

## Results

###  Characteristics Of Health Center Governing Boards 

 All 83 (100.0%) invited governing boards or their designees within the selected health centers, volunteered to be interviewed. Out of 83 respondents, 63 (75.9%) were board chairpersons and the remaining 20 (24.1%), were their designees. Table 2 presents the distribution of health centers enrolled in this study by region, zone and district. Slightly higher than one-third (34.0%) of the health centers were located in the Amhara region and 34.0% of health centers in this study were located in the Oromia region.

**Table 2:** Distribution of Targeted Health Centers by Number, Region, Zone and District, 2018

**Characteristics **	**Zones **		**Districts**		**Health Centers**	
**Region**	**No.**	**%**	**No.**	**%**	**No.**	**%**
Amhara	4	29%	6	33%	28	34%
Oromia	4	29%	4	22%	28	34%
SNNP	4	29%	4	22%	14	17%
Tigray	2	14%	4	22%	13	16%
Total	14	100%	18	100%	83	100%

Abbreviation: SNNP, Southern Nations, Nationalities, and People.

###  Structure Parameter of the Governing Boards 

 The average size of health center board members in the study area was 6.1, of which one of the members was female. Each health center had facilitated 5 meetings per annuum. Three-fourth (74.7%) of health center governing boards payed board members for every meeting they attended and participated in (Table 3). The payments ranged between US$2.5 to US$3.3 per meeting.

**Table 3:** Structure, Roles and Responsibilities, Training and Development-Related Scores of Health Center Governing Boards, 2018

**Characteristics**	**Amhara **	**Oromia **	**SNNP **	**Tigray **	**Overall **
**Structure **					
N (targets)	28	28	14	13	83
Mean number of board members	6.6	6.0	6.0	5.5	6.1
Mean number of female board members	0.46	1.00	1.14	1.76	0.96
Mean number of meetings per year	7.5	3.0	2.7	8.1	5.3
% Of boards that pay members per meeting	96.4	85.7	71.4	7.7	74.7
**Roles and responsibilities**					
% Of boards that review and approve annual plans	78.6	75.0	85.7	69.2	77.1
% Of boards that review and approve strategic plans	50.0	46.4	28.6	46.2	44.6
% Of boards that review and approve service reports	60.7	39.3	78.6	46.2	54.2
% Of boards that conduct a semi-annual performance evaluation of health center directors	67.9	64.3	71.4	69.2	67.5
% Of boards that facilitated multi-sectoral collaboration	21.4	14.3	57.1	30.8	26.5
% Of boards that facilitated reimbursement of credit services (CBHI)	78.6	71.4	100.0	84.6	80.7
% Of boards that facilitated loans of preservice service payments (CBHI)	64.3	71.4	71.4	38.5	63.9
% Of boards that approved budgets for quality improvement	67.9	64.3	57.1	53.8	62.7
% Of boards that approved budgets for staff training	21.4	17.9	28.6	23.1	21.7
% Of boards that review the quality of care on a quarterly basis or more frequently	60.7	57.1	57.1	30.8	54.2
% Of boards that review referral services	17.9	21.4	64.3	30.8	28.9
% Of boards that review patient complaints	60.7	28.6	92.9	46.3	53.0
% Of boards that review patient experiences	46.4	35.7	71.4	46.2	47.0
% Of boards that review community scorecards	28.6	14.3	42.9	46.2	28.9
% Of boards that organized community-facility interface meetings	50.0	10.7	92.9	30.8	41.0
**Training and development **					
% Of boards that have at least one member participate in governing board training programs	67.9	57.1	35.7	69.2	59.0
% Of boards that have all members participate in governing board training programs	39.3	28.6	21.4	38.5	32.5
% Of boards that need governing board training programs	57.1	71.4	78.6	53.8	65.1
% Of boards that have orientation manuals	67.9	57.1	42.9	53.8	57.8
**Mean overall governance score (out of 23)**	13.4	11.3	15.0	12.6	12.8

Abbreviation: CBHI, community-based health insurance.

###  Roles and Responsibilities of Governing Boards

 Slightly higher than three-fourth (77.1%) of health center governing boards had reviewed and approved their respective health center’s annual plans. The majority (80.7%) of health center governing boards were actively engaged in the reimbursement of pre-paid services delivered through a community-based health insurance (CBHI) scheme. More than two-thirds (67.5%) of health center governing boards had reviewed and appraised performances of health center directors on a semi-annual basis. Much lower than half (41.0%) of boards had organized community-facility interface meetings in the previous 6 months (Table 3).

###  Training and Development of Governing Boards

 Only 32.5% of health center governing boards offered health center governing board training programs to all members and less than two-thirds (59.0%) of those governing boards had at least one board member that participated in the training programs. Almost two-third (65.1%) of the health center governing boards stated that they have a need to participate in governing board training programs (Table 3).

###  Performance of Health Centers Against Minimum Standards 

**Figure 2:**
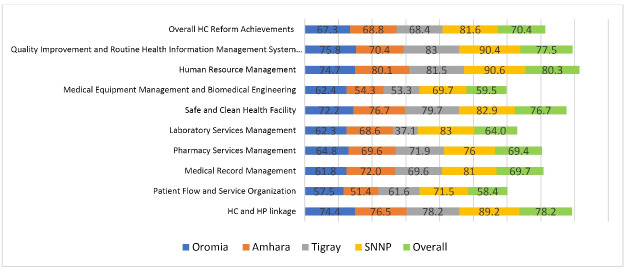
Stack Bar Presenting Performance of Health Centers Against Minimum Standards by Region, September 2018. The performance of health centers against the Ethiopian Health Center Reform Guidelines (EHCRIGs) presented by region and chapters using stack bars. Abbreviations: HC, Health Center; HP, Health Post.

 The overall EHCRIG performance score of the 83 health centers in this study was 70.4% (SD ± 15.0%). The highest 3 scores observed were 80.3%, 78.2%, and 77.5% for human resource management, health center and health post linkage and quality improvement and routine health information system, respectively. The 3 lowest scoring chapters were 58.4% 59.5%, and 64.0% for patient flow and service organization, medical equipment management and biomedical engineering, and laboratory service management respectively (Figure 2).

###  Governing Board Functionalities and Performance Categorization of Health Centers 

 Health center governing board functionalities and performance of health centers were divided into 3 categories. The mean health center governing board function score with standard deviation was 56.0% (SD ± 14.5%). Only 5, (4.8) of the health center governing boards scored greater or equal to 80% and were categorized under the ‘functional’ governing board category. 23 (27.7%) of health centers scored greater or equal to 80.0% on EHCRIG minimum reform standards and were categorized as ‘high’ performing health centers (Table 4).

**Table 4:** Categorization of Governing Board Functionalities and Health Centers’ Reform Performance, 2018

**Functionality Category of Governing Boards**		**Performance Category of Health Centers **	
**Criteria **	**No. (%)**	**Criteria (reform)**	**No. (%)**
Functional (≥80%)	4 (4.8%)	High (≥80%)	23 (27.7%)
Semi-functional (60%–79.9%)	32 (38.6%)	Medium (60%–79.9%)	45 (54.2%)
Non-functional (<60%)	47 (56.6%)	Low <60%	15 (18.2%)

###  Governance and Health Center Standards Met – Spearman’s Correlation Analysis

 The performance and functionality scores of health center governing boards were computed for correlation analysis. Structure, (rho = 0.312), and roles and responsibilities, (rho = 0.916) scores had a statistically significant positive correlation with overall health center governance scores at *P* < .001. Similarly, there was a statistically significant strong positive correlation among health center governing boards’ roles and responsibilities scores, (rho = 0.866), overall governance scores, (rho = 0.828) and health center reform performance (EHCRIG) scores at *P* < .001 (Table 5).

**Table 5:** Spearman’s Correlation Analysis of the 4 Parameters: Structure, Roles and Responsibilities, Training and Development and Performance of Health Centers, 2018

**Correlations**						
**Parameters **		**(1)**	**(2)**	**(3)**	**(4)**	**(5) **
Structure (1)	Spearman's correlation coefficient	1.000	0.048	0.115	0.312^a^	0.012
	Sig. (2-tailed)	-	.665	.299	.004	.918
	N	83	83	83	83	83
Roles and responsibilities (2)	Spearman's correlation coefficient	0.048	1.000	-0.090	0.916^a^	0.866^a^
	Sig. (2-tailed)	.665	-	.418	.000	.000
	N	83	83	83	83	83
Training and development (3)	Spearman's correlation coefficient	0.115	-0.090	1.000	0.194	0.000
	Sig. (2-tailed)	.299	.418	-	.079	.997
	N	83	83	83	83	83
Overall governance score (4)	Spearman's correlation coefficient	0.312^a^	0.916^a^	0.194	1.000	0.828^a^
	Sig. (2-tailed)	.004	.000	.079	-	.000
	N	83	83	83	83	83
Health center performance standards met (5)	Spearman's correlation coefficient	0.012	0.866^a^	0.000	0.828^a^	1.000
	Sig. (2-tailed)	.918	.000	.997	.000	-
	N	83	83	83	83	83

^a^Correlation is significant at the.01 level (2-tailed).
Perfect: If the value is near ±1, then it said to be a perfect correlation; as one variable increases, the other variable tends to also increase (if positive) or decrease (if negative). High degree: If the coefficient value lies between ± 0.50 and ± 1, then it is said to be a strong correlation. Moderate degree: If the value lies between ±0.30 and ±0.49, then it is said to be a medium correlation. Low degree: When the value lies below +0.29, then it is said to be a small correlation. No correlation: When the value is zero.^22^

## Discussion

 This research attempted to assess the relationship between the functionality of health center governing boards and the performance of health centers, against minimum standards set in health center reform guidelines in Ethiopia. All invited governing board chairs, or their designees participated in the study. The findings of this study provide scientific evidence on the relationship between the functionality of governing boards and clinical and management related performance of health centers in Ethiopia.

 The theoretical model used to analyze the findings suggested that employing a measurement standard is a first step in assessing performance management. This study noted that the national level measurement standards have been rolled out to the lower levels of the health system as all the assessed facilities are measuring their performances against those standards. This finding was in line with Kringos et al who confer that the first step in performance management should be a comprehension of relevant indicators by the relevant decision-makers.^17^ Similarly, Smith et al underpin the importance of setting measurement indicators and implementing performance management as an initial step towards ensuring the health system’s continuous improvement and accountability.^23^

 The second step of the performance intelligence framework is the translation of healthcare data and indicators into knowledge and information which can be used for governance and management. In addition to processing of data as part of the health system management, having a functional structure that conveys the processed information and drives the required changes is an important segment of the model. Thus, in Ethiopia, the functionality of the governance structure – the health center board – is particularly important to ensure this step is well-implemented. The functionality of governing boards was assessed in terms of structure, training and development, and roles and responsibilities.

 This study documented that there were 5 to 7 members employed in each board and the gender mix was observed to be below the recommendations of the health reform standard. This finding was in line with a report by McNatt et al on the number of hospital governing boards which ranged from 5 to 7.^19^ In addition, one-third of the governing boards did not have any member that had attended a governing board training program. Training programs help board members to have a clear understanding of policies, guidelines, projects and program management skills.^11^ As Martineau et al confirm, enhancing managerial competencies help the health system to achieve better results.^24^ Similarly, Fusheini et al explored the necessary skills required for governing boards, namely, planning, monitoring and financial management for improved performances.^25^ The reported lack of board member participation in training and development programs might be attributed to the high level of turnover of sector office heads.

 The utilization of the knowledge by all stakeholders, the third segment of the model, is also crucial to driving changes in performance. Knowledge and information generated helps all stakeholders develop several agreed performance improvement action plans. Therefore, the utilization of locally generated knowledge and information at the point of production through doable actions should be the responsibility of all stakeholders at primary healthcare entities.^15-17^ These findings were in line with Argaw et al who documented that working against minimum standards and developing clear and doable action plans can improve the performance scores of primary healthcare entities.^12^

 A well-functioning health center governing board which achieves high scores against minimum standards contributes to an increased number of high performing health centers. These significant, positive results can be achieved through enhancing the capacity of health center management teams in attaining minimum standards.^12^ This was in line with the findings of Dhaba et al and Linnander et al, who reported that healthcare professional capacity development will contribute to high performing health systems and can help achieve favorable results in hospital reform in Ethiopia.^26-28^ This study found the presence of a statistically significant and strong correlation (Spearman rho correlation coefficient) between health center performance standard high scores on roles and responsibilities of governing board parameters (rho = 0.866) and overall governance scores (rho = 0.828). In addition, the study found a moderate correlation between health center performance and structure parameters of governing boards. Hence, providing technical support to health center governing boards to execute their major roles and responsibilities should get adequate emphasis to bring about the desired results. This finding is consistent with the positive association between governing board function and performance of primary hospitals in Ethiopia documented by McNatt et al.^26^

###  Limitations of the Study

 This study has some known limitations. Like many cross-sectional studies, it is difficult to determine causal relationships. In addition, to reduce the effects of secondary data on missing important third variables, primary data were collected from board chairs or their designees. The sample size of 83 can be considered too small to generalize the findings. Nevertheless, the results can be used for performance and quality improvement interventions in the study areas and other low-income countries.

## Conclusions and Recommendations

 Based on the results of this study, it can be concluded that a well-functioning health center governing board can improve health center clinical and management standard adherence. Well-functioning health center governing boards regularly review and approve plans, monitor the performance of health center directors, mobilize resources and allocate budgets for quality improvement. Therefore, continuing to strengthen the capacity of governing boards, focusing on improving implementation of their roles and responsibilities, and continuing training and development on finance and business management is recommended.

## Acknowledgements

 The investigators are indebted to all participants for their cooperation during data collection. This technical report on governing boards and performance of health centers is made possible by the generous support of the American people through USAID. The authors thank Heran Demissie and Ismael Ali for English language copy editing and study site map development, respectively.

## Ethical issues

 The research protocol was reviewed and ethical clearance was obtained from the Amhara Public Health Institute (Ref. No. HRTT02/137/2018), the Oromia Regional State Health Bureau (Ref. No. BEFO/HBTPH/1-8/476), the SNNP Regional State Health Bureau (Ref. No. PLMG-19/8407) and the Tigray Regional State Health Bureau (Ref. No. 453/1418/10) Institution Review Boards and Research Ethics Committees. In this study, no facility identifier information was collected. Oral and written consent was obtained from all research participants. The study had no known risk and no payment was made to participants. The anonymous data were stored in a password locked computer. The USAID Transform Primary Healthcare Activity submitted summary reports on a quarterly basis to district health offices, zone health departments and regional health bureaus. Hence, the information was used for performance improvement.

## Competing interests

 Authors declare that they have no competing interests.

## Authors’ contributions

 MDA and BFD equally made a substantial contribution to conceiving and designing the study, and MDA was responsible for overseeing the field work, cleaning the data, analysing the data, interpreting the analysis and drafting the manuscript. Both authors read the final document and approved it. MDA, the corresponding author, submitted the manuscript for publication.

## Disclaimer

 The authors’ views expressed in this technical report do not necessarily reflect the views of USAID or the United States Government.

## Funding

 Transform Primary Health Care project is a United States Agency for International Development (USAID) funded health program under cooperative agreement number of AID-663-A-17-00002. The program is implemented by a consortium of organizations which includes Pathfinder International, JSI Research and Training Institute, Inc., EnCompass, Malaria Consortium, Abt Associate Inc., and Ethiopian Midwifes Association in collaboration with local Government and Non-government partners. The funder does not have any role in the design of this study, data collection, analysis and writing of the manuscript.

## Endnotes

 [1] EHCRIGs are a set of minimum clinical and management standards which all health centers in Ethiopia required to adhere to. [2] USAID Transform: Primary Healthcare Activity is a bilateral project, implemented by consortium of international, local development partners in collaboration with communities and the public health sector in 4 regional states of Ethiopia. [3] Health center governing boards are established through regional state health bureaus directives. The members include representatives of the community, health workers, and sector office heads.

## Supplementary files

Supplementary file 1. Annexure - Governing Board.

Supplementary file 2. Content of Governing Board Training Program.
