# Supplementary material for: Examining Governing Board Functions and Health Center Performances During Health System Reform: A Cross-sectional Study in 4 Regional States of Ethiopia
Source: Int J Health Policy Manag. 2020 Dec 2;11(7):928–36. doi: 10.34172/ijhpm.2020.235 (PMC9808206; doi:10.34172/ijhpm.2020.235)
Supplement: Supplementary file 1 — Annexure - Governing Board. [file ijhpm-11-928-s001.pdf]

Supplementary file 1

Annexure - governing board

Introduction

This rapid assessment is designed to document the function of health center governing boards and investigate the association between its role and health center performances. USAID Transform: Primary Health Care will analyze the data and synthesize the information to advocate for best strategies to improve access to quality and equitable health services.

The tool has four parts. Part one is dedicated to socio-demographic characteristics of facilitators, part two is dedicated to information on the role of governing boards, part three is dedicated to capture the opinions of chairs or designees on their governing boards, and part four is dedicated t extract data on health reform performances. Thank you so much for taking part.

**Health Center Governance Assessment**		
**SN**	**Question**	**Coding Categories**
**I**	**Basic Information**	
1.1	Date of assessment	__ __/__ __/__ __ __ __(DD/MM/YYYY)
1.2	Region	
1.3	Zone	
1.4	Woreda	
1.5	Organization name	
1.6	Location of health center	1. Urban<br>2. Rural<br>3. Others, specify______________________
1.7	Respondent details	1. Health center board chair<br>2. Designee of the health center governing board<br>3. Others, specify______________________
1.8	Number of board members (male & female)	

**Health Center Governance Assessment**		
1.9	Number of female board members	
1.10	Number of board meetings per year	
1.10.1	Write the four previous meeting agendas and date	Meeting 1:<br>Date:<br>Meeting 2:<br>Date<br>Meeting 3:<br>Date<br>Meeting 4:<br>Date
1.11	Does the health center pay board members during business meetings?	1. Yes<br>2. No
**2**	**Roles and responsibilities**	
2.1.	Does the board approve an annual workplan?	1. Yes 2. No
2.2.	Does the board develop and approve its strategic plan of the health center (new revenue, services)?	1. Yes 2. No
2.3.	Does the board approve service reports?	1. Yes 2. No
2.4.	Does the board conduct performance evaluations of the health center director on a semi-annual basis?	1. Yes 2. No
2.5.	Does the board facilitate intersectoral collaboration (with education, water and energy, women & child affairs, finance and economy offices.)?<br>Collect: action plans, activity reports and initiatives	1. Yes 2. No<br>3). If the answer is Yes, "take detailed note."
2.6.	Does the board facilitate the reimbursement of post services from the Community Based Health Insurance scheme?	1. Yes 2. No
2.7.	Does the board facilitate loans (pre—service advance) to health center from the Community Based Health Insurance scheme?	1. Yes 2. No

Health Center Governance Assessment		
2.8.	Does the board approve budgets for quality improvement from retained revenues?	1.Yes 2. No
2.9.	Does the board approve budgets for staff training?	1. Yes 2. No
2.10.	Does the board review quality of care on a quarterly basis or more often?	1. Yes 2. No
2.11.	Does the board review referral services?	1. Yes 2. No
2.12.	Does board review patient complaints?	1. Yes 2. No
2.13.	Does the board review patient/client experience on a quarterly basis or more often?	1. Yes 2. No
2.14.	Does the board review community scorecards?<br>Does the board facilitate health center community interface meetings (town hall meetings)?	1. Yes 2. No
**3**	**Training and orientation**	
3.1.	Do all members of the board receive training and orientation on business and financial management?	1. Yes 2. No
3.2.	Do all board members need training on business/financial management?	1. Yes 2. No
3.3.	Does the board have orientation manuals?	1. Yes 2. No

Open ended question

"Please share any other comments or concerns you have in relation to the functioning of your governing board?"

___

___

___

___

___

___

___

Data abstraction form

Ser. No.	Chapter description	Percentage (%)
1	HC and HP linkage	
2	Patient Flow and Service Organization	
3	Medical Record Management	
4	Pharmacy Services Management	
5	Laboratory Services Management	
6	Safe and Clean Health Facility	
7	Medical Equipment Management and Biomedical Engineering	
8	Human Resource Management	
9	Quality Improvement and Routine Health Information Management System Management	
10	Overall HC Reform Achievements	
