# Supplementary material for: Examining Governing Board Functions and Health Center Performances During Health System Reform: A Cross-sectional Study in 4 Regional States of Ethiopia
Source: Int J Health Policy Manag. 2020 Dec 2;11(7):928–36. doi: 10.34172/ijhpm.2020.235 (PMC9808206; doi:10.34172/ijhpm.2020.235)
Supplement: Supplementary file 2 — Content of Governing Board Training Program. [file ijhpm-11-928-s002.pdf]

Supplementary file 2

Content of governing board training program

I. Governance
   a. What is governance?
   b. What are the district council expectation of governing boards?
   c. Roles and responsibilities of governing board
   d. Jurisdiction and power of health center governing board
   e. Leadership and code of conduct of governing board members
   f. Role of chairman, members and health center director
   g. Disclosure of gifts and loans
   h. Conflict of interest
   i. Meeting agendas and rules
   j. Policies, guidelines ad protocols
   k. Health center committees
   l. Complaints management
   m. Adapting code of conduct
   n. Public interest vs private interest
   o. Dealing with misconduct

II. Performance monitoring
   a. Criteria for doing good job
   b. Health centers reporting system
   c. Benchmarking

III. Patient and community involvement
   a. Patient rights and responsibilities
   b. Involving community
   c. Community scorecards

IV. Business and financial management
   a. Planning cycle
   b. Health center plan

c. Health center operational business plan
d. Annual budget
e. Annual report
f. Revenue and expenditure
g. Raising revenue
h. Commercial activity
i. Fees and charges
j. Grant and subsidies
k. Borrowings

Thank you so much!
